# Task-shifting to improve asthma education for Malawian children: a qualitative analysis

**DOI:** 10.1186/s12960-021-00576-1

**Published:** 2021-03-02

**Authors:** Lovemore Nkhalamba, Sarah Rylance, Adamson S. Muula, Kevin Mortimer, Felix Limbani

**Affiliations:** 1 Lung Health Group, Malawi-Liverpool-Wellcome Trust Clinical Research Programme, P.O. Box 30096, Blantyre 3, Malawi; 2 Department of Clinical Sciences, Liverpool School of Tropical Medicine, Liverpool, UK; 3 Department of Public Health, College of Medicine, University of Malawi, Blantyre, Malawi

**Keywords:** Asthma, Task-shifting, Sub-Saharan Africa, Children, Lay educators

## Abstract

**Background:**

Asthma education, a key component of long-term asthma management, is challenging in resource-limited settings with shortages of clinical staff. Task-shifting educational roles to lay (non-clinical) staff is a potential solution. We conducted a randomised controlled trial of an enhanced asthma care intervention for children in Malawi, which included reallocation of asthma education tasks to lay-educators. In this qualitative sub-study, we explored the experiences of asthmatic children, their families and lay-educators, to assess the acceptability, facilitators and barriers, and perceived value of the task-shifting asthma education intervention.

**Methods:**

We conducted six focus group discussions, including 15 children and 28 carers, and individual interviews with four lay-educators and a senior nurse. Translated transcripts were coded independently by three researchers and key themes identified.

**Results:**

Prior to the intervention, participants reported challenges in asthma care including the busy and sometimes hostile clinical environment, lack of access to information and the erratic supply of medication. The education sessions were well received: participants reported greater understanding of asthma and their treatment and confidence to manage symptoms. The lay-educators appreciated pre-intervention training, written guidelines, and access to clinical support. Low education levels among carers presented challenges, requiring an open, non-critical and individualised approach.

**Discussion:**

Asthma education can be successfully delivered by lay-educators with adequate training, supervision and support, with benefits to the patients, their families and the community. Wider implementation could help address human resource shortages and support progress towards Universal Health Coverage.

*Trial registration* The RCT was registered in the Pan African Clinical Trials Registry: PACTR201807211617031

**Supplementary Information:**

The online version contains supplementary material available at 10.1186/s12960-021-00576-1.

## Introduction

Asthma is a growing public health burden in low- and middle-income countries (LMICs); worldwide, approximately 1000 people die every day, with the majority of deaths occurring in LMICs [1]. Asthma is the most common chronic disease among children and is a major cause of morbidity [2]. Limited data suggest that asthma prevalence is increasing among children in African countries, with those affected suffering more severe symptoms than children from high-income settings [3]. There are no published asthma prevalence data for Malawi. Children with acute asthma attacks frequently attend for emergency care at the country’s largest tertiary hospital, Queen Elizabeth Central Hospital (QECH): severe cases require hospital admission with considerable associated costs for the child, family and health system.

Achieving good symptom control is a primary goal in asthma management and adherence to treatment plays a key role [4]. Adherence is enhanced if the family has a positive view of asthma, understand the need for regular inhalers and trust the medication [5]. During childhood, parents or carers are primarily responsible for medication administration, identification and avoidance of triggers and obtaining prescriptions [6]. However, older children can take an increasingly active role is self-management of their asthma [7]. Successful asthma care then requires that both children and parents receive adequate information on asthma, triggers, medication and self-management of symptoms.

Asthma education is central to the Global Initiative for Asthma (GINA) recommendations, which emphasize the importance of a strong partnership between patients and health care providers [4]. Provision of asthma education to children and their families reduce the mean number of hospitalizations and emergency room visits in high income countries (HICs) [8].

Asthma care in LMICs is often delivered in overburdened health care settings, and health care providers may have little time to spend on asthma education. Task-shifting, defined as “the transfer of tasks normally performed by a more to a less qualified cadre with a different level of education and training, or to a person specifically trained to perform a limited task only, without professional education”, has been suggested as an effective and affordable strategy to improve the management of non-communicable diseases in LMICs [9]. However, there are limited data on task-shifting in asthma care in LMICs, and the use of non-clinical personnel has not been explored [10]. Considering this, we designed an intervention to improve care at a tertiary hospital in Malawi, including individualised asthma education delivered by non-clinical staff [11].

In this qualitative sub-study, the overarching research question was ‘is delivering asthma education using non-medical staff feasible and acceptable in our setting?’ To answer this question, we aimed to explore the experiences of the asthma educators, the patients and their carers participating in the asthma education sessions, in order to: (1) assess the acceptability of using non-clinical staff to deliver asthma education; (2) understand facilitators and barriers to asthma education; (3) assess the perceived value of the education sessions to children and their carers.

## Methods

### The asthma education intervention

This qualitative sub-study was part of a randomised-controlled trial (RCT), recruiting children aged 6–15 years with a doctor-diagnosis of asthma from the paediatric outpatient clinic at Queen Elizabeth Central Hospital (QECH), a tertiary government hospital in Blantyre, Malawi (Pan African Clinical Trials Registry reference PACTR201807211617031) [11]. Participants and their carers attended an asthma education session, delivered by non-clinical staff (“asthma educators”) as part of the study intervention. The asthma education session followed a standardised approach, with educators completing a checklist to ensure consistency within and between study personnel. Asthma education was reinforced at subsequent study visits by the educators. As part of the study team, a senior research nurse provided the educators with daily clinical support and complicated cases were discussed with a consultant paediatrician (SR). The asthma educators were individuals with no clinical training but had completed at least 12 years of formal education (primary and secondary education) and attained the Malawi School Certificate of Education (MSCE). Prior to study initiation, the educators underwent a structured training programme, designed and delivered by a consultant paediatrician (SR), including clinic observations, tutorials and role play (see Additional file 1).

In total, 120 participants were recruited to the RCT between September 2018 and December 2019; the qualitative assessment of the asthma education intervention was conducted between August 2019 and March 2020.

### Study site

QECH, a tertiary-level, government referral hospital is located in Blantyre, the second largest city in Malawi, in the Southern region. Outpatient asthma care at QECH is usually provided in a busy, under-staffed general paediatric clinic, with little time for assessing asthma control or providing asthma education. Primary health care in Malawi is offered at health centres within communities. Although some health centres within Blantyre city review asthma patients, inhaled medication is mostly unavailable and patients are referred to QECH.

### Study design

The sub-study used qualitative research methods including focus group discussions (FGDs) with study participants and their carers and in-depth interviews with study staff (Table 1) [12]. We incorporated a phenomenological approach to help us understand lived experiences and people’s perspective of the asthma education and their interpretation more inductively. We interviewed all the study staff who were involved with the intervention: four lay educators and the senior nurse who supervised them. The children and carers that attended the FGDs were purposively sampled and we continued to conduct the FGDs until saturation was reached. These aimed at gaining deeper insight into the facilitators and barriers to the educational aspect of the intervention.

**Table 1:** Qualitative study participants, attending focus group discussions and interviews

Method	Number of participants	No. of contacts	Data collected
*Focus Group Discussions (FGDs)*			
Mothers and other female carers	21	3 FGDs	Exploring the children’s and carers’ experience of the asthma education including barriers and facilitators to the intervention, their perceptions of the asthma educators as non-medical staff delivering the asthma education, their perceived value of the asthma education and recommendations for future interventions
Fathers and other male carers	7	1 FGD	
Children	15	2 FGDs	
Total FGD participants	43		
*In depth interviews*			
Asthma educators	4	4 interviews	Asthma educators’ experience of delivering the asthma education, their training and other mechanisms for support, perceived benefits to the children, facilitators and barriers to delivery and recommendations
Research nurse	1	1 interview	Exploring her experience in supervising the asthma educators and the barriers or facilitators to delivery, uptake of the asthma education and recommendations
Total interview participants	5		

#### Data collection

FGDs and interviews were conducted in Chichewa by a research assistant (LN), a native Chichewa speaker, trained and certified in qualitative research methods, who was separate to the RCT study team. The discussions were led using semi-structured topic guides and recordings were transcribed verbatim and translated into English for further analysis.

#### Eligibility criteria

Participants were approached following completion of the RCT intervention. Children (aged ≥ 10 years) and their carers were purposively sampled to ensure that different key characteristics were represented: age, sex, and asthma severity. Only parents or carers who attended the asthma education sessions were included. Due to Malawian cultural norms, men often dominate discussions: male and female carers were therefore invited to attend separate FGDs to encourage free participation.

Individual interviews were conducted with each of the “asthma educators” and with the research nurse who had supervised the sessions. Interviews took place in a private location, with full confidentiality assured to encourage honest and open participation.

### Data analysis

Data analysis was conducted iteratively alongside data collection, to allow exploration of emerging issues in later interviews and FGDs. A thematic approach was adopted, with all transcripts coded independently by three authors (LN, SR and FL) manually and later compared to enhance the rigour of the results. We also triangulated our data by considering perspectives and experiences from the carers, children and the educators to further enhance the validity and rigour of our findings. A coding framework was developed by the authors (LN, SR and FL) which was used to code all the transcripts through identification of informative texts and quotations [13]. The codes were grouped into key themes derived from study objectives (deductively) and emerging from the transcripts (inductively) [14].

## Results

Four key themes emerged from the FGDs and in-depth interviews and are discussed in the following sub-sections; (1) challenges with asthma care in Blantyre; (2) acceptability of using non-clinicians as educators; (3) perceived value of asthma education sessions; (4) facilitators and barriers to delivery and uptake of asthma education, including recommendations.

### Challenges with asthma care in Blantyre

#### Busy clinical environment

Participants reported several challenges in accessing asthma care within the government health facilities. Both parents and children commented that health care workers did not have enough time to explain the various aspects of asthma management, both during admissions and outpatient attendances, largely due to the busy clinical environment. Parents felt unable to ask all the questions they had about their child’s medication and asthma more generally.

*“…the explanation there is really brief, and you will be lucky if you find a person that is able to answer any question that you have because they are very busy.” Mother of 12-year-old asthmatic child, FGD.*

In addition, some parents and children also expressed their concerns about hostile attitudes they had encountered from some medical staff previously, which affected their willingness to ask for clarification when needed. Children also reported they were given conflicting information from different doctors, which was confusing.

*“Some doctors get really angry and annoyed when you keep asking questions.” Mother of 11-year-old asthmatic child, FGD.*

*“We kept on meeting different doctors at the clinic, that was really disturbing me because you end up being told different things by different people.” 13-year-old male asthmatic, FGD.*

#### Access to information

Another challenge reported by both parents and children was the lack of asthma information provided by health care staff. Some parents expressed their lack of knowledge of what the disease (asthma) is and how it affects the human body. Specific areas of concern were what to do during an asthma attack and how to administer inhaled medication. Children said they were not aware of the triggers for their asthma or the importance of using inhaled treatments.

*“In fact, I didn’t even know that asthma causes the airways to close but when we came here, they started teaching us from there” Father of 7-year-old child, FGD.*

#### Access to medication

Access to inhaled medication was also expressed as a challenge, especially by parents, with inhaled medication largely unavailable at primary health centres. Parents described extremely stressful situations when they had no medication to use at home during a severe attack.

*“She was attacked at around 10 in the night, we didn’t have an inhaler. So, we tried making phone calls to try and find an inhaler from other people, but we didn’t find it. And then we tried looking for transport, we still didn’t find it. We were only able to get to the hospital at 4 in the morning.” Father of 14-year-old asthmatic child, FGD.*

### Acceptability of using non-clinicians as educators

#### Perspectives of patients and families

The parents did not express any concerns that the education sessions were delivered by non-clinical personnel. Some of the parents said they assumed the educators had some medical training because of their professional manner. Many parents praised the educators’ overall competence and asthma-related expertise. The children said the asthma educators were friendly and caring and that they felt free to ask questions without fear of being rebuked. Parents also said the openness and friendliness of the educators made the children look forward to coming back for the next study appointment.

#### Perspectives of the asthma educators

The asthma educators said they were initially nervous to conduct the asthma education sessions with patients and their families. They reported that their knowledge on asthma was very limited before they participated in the training, after which they understood more about the disease and how to deliver the sessions. The educators also commented that they gained confidence to deliver the education sessions over time, with ongoing experience.

### Perceived value of asthma education

Participants described how various aspects of their lives were before the asthma education intervention and the subsequent impact of the education they had received (Table 2).

**Table 2:** Participant’s perceptions of the impact of asthma education on knowledge levels, symptom control and aspects of daily life

Before asthma education intervention	After asthma education intervention
*Participants’ reports of asthma knowledge*	
No clear understanding of asthma, common triggers and inhaled medications Unable to identify asthma symptoms	Improved knowledge of asthma, common triggers and inhaled medications Greater understanding of what to do in an emergency Confidence to identify symptoms of asthma and manage appropriately
*Participants’ reports of asthma symptoms*	
Difficulties breathing at night, often interfering with sleep Frequent cough and wheeze Frequent visits to health facilities Frequent school absence	Families able to manage asthma symptoms more effectively Fewer attacks, school absence and hospital visits
*Interaction between asthma and family life*	
Disruption to sleep for whole family Stressful situations during deteriorating symptoms Staying home to care for child Removing child from school to allow closer monitoring	Greater control of asthma Knowledge of asthma triggers and self-management has reduced child’s symptoms and enabled parents to be more productive Improved asthma knowledge among wider family, including other asthmatic individuals
*Interaction between asthma and school life*	
Stigmatised by peers Lack of understanding among school community Belief that asthma is contagious	Children gaining support from peers through greater openness and understanding
*Interaction between asthma and the community*	
Negative attitudes towards inhaled treatment Belief in healing through traditional medicines and prayers	Parents keen to act as asthma advocates and share their new knowledge with the wider community

Children who were frequently sick and often missed school due to their asthma described a great improvement since implementing what they had learnt during the asthma education sessions. The improvement in clinical condition had a positive impact on family daily life, with families reporting reduced school absence and increased productivity at work.

*“When it’s time for me to go to the village to farm, seeing that she won't be able to stay without being monitored, I was withdrawing her from her school here in town and I would go with her to the village. But all that stopped now - I am able to leave her.” Mother of 13-year-old asthmatic child, FGD.*

Parents were previously anxious about how to manage their child’s asthma, particularly during an attack, and how to use their medication but reported increased confidence and a feeling of control, as a result of their increased knowledge levels.

*“Most times…. when the child starts to get sick, we would not do anything. We would wait till maybe two days pass and then start off to the hospital. But when we were taught, it really helped…. When he gets sick again, before it reaches the point of taking him to the hospital, because of what we learnt in the research we are able to help him control the asthma before it gets worse.” Mother of 7-year-old asthmatic child, FGD*

Both parents and children reported misconceptions and negative opinions relating to asthma and inhaled medication which they had experienced from family members, school peers and the wider community.

*“When I am at school and I have asthma symptoms, my friends tell me that I am bewitched and when I am trying to play with them, they tell me that I will spread my asthma to them.” 11-year-old male Child, FGD.*

*“Some of my friends scared me saying "That is a bad drug, if your child starts using inhalers now, his asthma will never improve and will be dependent on inhalers all his life." I was really scared so much that when I got home with him, I didn’t use the inhalers, I just kept them.” Mother of 6-year-old child, FGD.*

### Facilitators and barriers to asthma education

#### Intervention design: training, guidelines and support

The asthma educators and the study nurse mentioned specific resources which were helpful in ensuring the education sessions were delivered effectively. The asthma educators explained that the pre-study training they received was one of the main activities that helped them gain knowledge and confidence to deliver the sessions effectively. The educators also reported that education session checklist ensured that everyone was teaching information uniformly, was helpful in reminding the educators of their own training, and helped staff to focus while teaching the participants.

Both the educators and the study nurse mentioned that the support given by supervisors and peers was also essential in ensuring the educators delivered the sessions effectively. The study nurse reported that she was available to the educators to help answer any questions and provide any additional support as needed. The educators also described the positive and motivating effect of words of appreciation from the study participants.

#### Individual and open approach to education sessions

Both children and their carers reported that the asthma educators were very approachable, patient and friendly which helped in understanding the asthma education sessions. The parents also said the educators ensured they felt comfortable to ask any questions that they had about their child’s condition or asthma more generally. The educators described the importance of building a good rapport with their patients when meeting them for the first time as this ensured that everyone was open and free to learn and ask questions.

*“First of all, we build a rapport in order to create an environment for both participant and guardian to feel that they are free…. they shouldn’t be afraid of anything.” Asthma educator, interview.*

Asthma educators said the difference in levels of education of the parents was one of the main barriers to the delivery of asthma education. Although the session was delivered in Chichewa, which parents appreciated, some still found it challenging to follow the content.

The educators explained that due to different baseline education levels among parents and children, they made sure that information was delivered according to an individual’s ability to understand and paused frequently to check comprehension and give clarification. Parents appreciated the physical demonstration of inhaler administration techniques, using an improved “bottle spacer”.

#### Recommendations

The parents and the children reported recommendations that can be put in place to overcome some of the barriers to delivery and uptake of the asthma education that they experienced:

1. Both the parents and children recommended that asthma education sessions should be conducted in a private and well-sheltered location.
2. Some of the children reported that the education sessions interrupted their school schedule, and that this was problematic—however, others commented that this disruption was acceptable, due to the beneficial nature of the education sessions.
3. Some parents highlighted the importance of providing additional written information, to reinforce the asthma education they had received. Written information would allow participants to revisit the information at a later date and also help share the knowledge with the wider community.

**Fig. 1:**
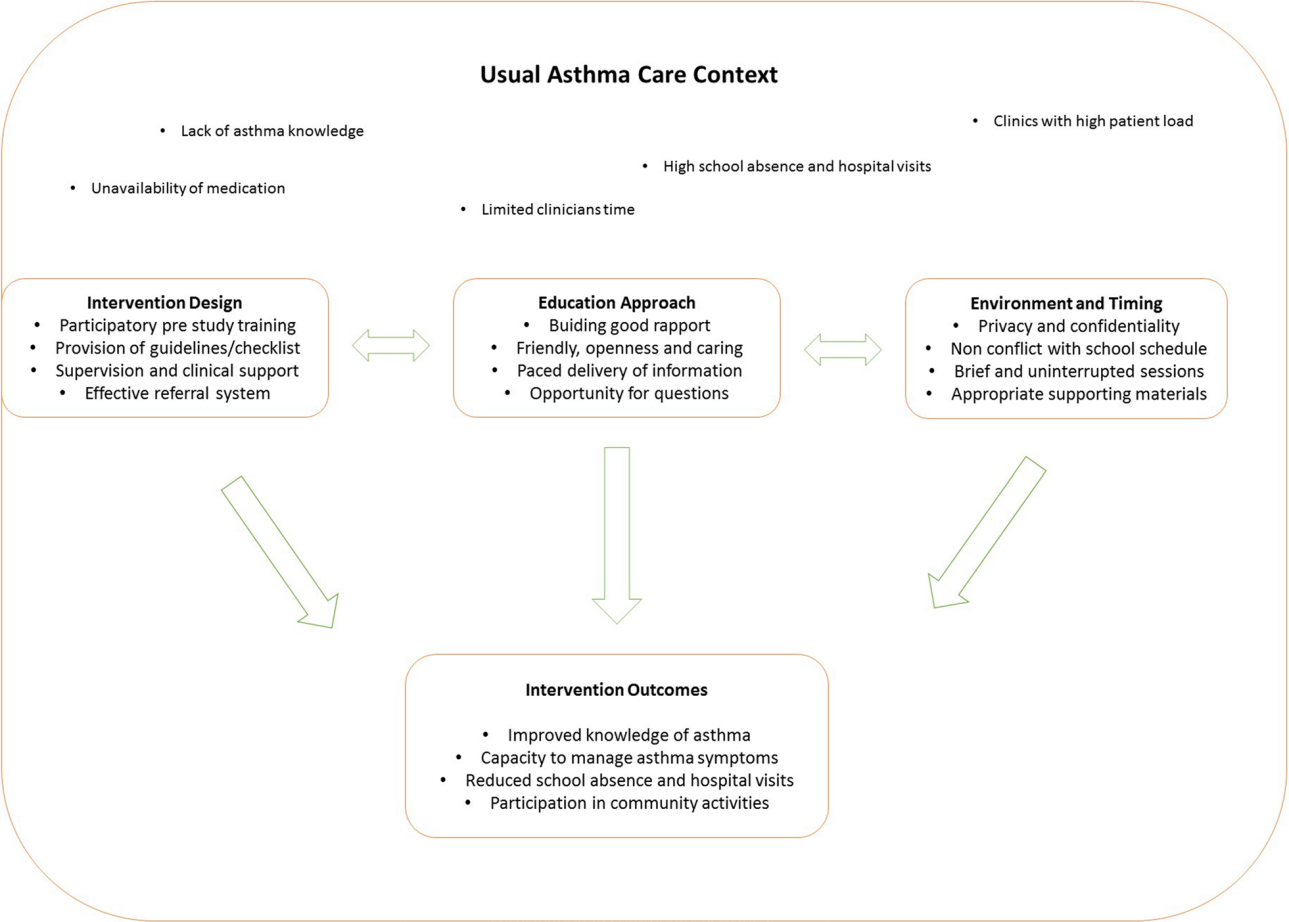
Task-shifting of asthma education to lay educators: dimensions of an ideal intervention

We collated the dimensions of our intervention and the context in which it was delivered in to show the key components of an ideal task-shifting intervention for asthma education by lay educators (Fig. 1).

## Discussion

To our knowledge this is the first evaluation of an asthma education task-shifting intervention in Sub-Saharan Africa (SSA), in which asthma education is delivered by educators with no medical or nursing background. Patients and their parents expressed high levels of satisfaction and described the positive impact of asthma education on their knowledge levels, asthma symptoms and daily life. Families reported increased confidence to self-manage asthma attacks at home, resulting in reduced absence from school and work, and fewer emergency health facility attendances. The educators emphasized the importance of building a good rapport with the patient and their family, and pacing the delivery of information, considering the participants’ background educational level. Young people and their families appreciated the open and friendly approach of the educators, and the time and patience that were taken to ensure understanding and address all their questions. We found that asthma education can be delivered successfully by non-medical personnel, given adequate training, and ongoing support from clinical staff, and that this approach was popular with young people and their families.

Task shifting is an attractive strategy in a resource-limited setting such as Malawi. The shortage of health workforce in LMICs is a major obstacle to the delivery of good quality care for chronic non-communicable diseases (NCDs) [9]. The WHO defines critical staffing shortage if a country has fewer than 2.5 health service providers (doctors, nurses and midwives) per 1000 population; 36 of the 57 countries identified as such are in SSA, with Malawi reporting two physicians and 28 nursing and midwifery personnel per 100,000 population in 2016 [15]. Although task-shifting has been evaluated for several NCDs, the evidence for asthma is scarce [9, 16]. One study in rural Cameroon reported improved outcomes for patients who received nurse-led asthma care, although 40% of patients had no follow-up data [10].

In Malawi, task-shifting of HIV screening to non-medical cadres has been successfully deployed with lay counsellors delivering HIV counselling and testing with good programme outcomes [17]. Health Surveillance Assistants (non-clinician health workers) have also been employed to deliver community case management of childhood illnesses; in common with our findings and others, the importance of ongoing support and supervision were highlighted in an evaluation of this scheme [18, 19]. Also in common with our study, recognition by the community and positive feedback were also mentioned as motivating factors [20].

In high-income settings, a small number of studies have evaluated peer- and lay-led complex asthma interventions for adolescents, suggesting a small improvement in asthma-related quality of life, although the effect on asthma control, exacerbations and adherence are unclear [21]. Self-management education delivered to adults with asthma by trained lay people, resulted in comparable clinical outcomes to patients seen by primary care based practice nurses in the UK [22]. Qualitative exploration of the experiences of these lay educators reinforced several of the points raised in our study; the need for comprehensive support and monitoring, particularly at the start of the programme, and the importance of training, with consideration of content, intensity, and interactive teaching methods [23].

In the USA, asthma education delivered by trained lay volunteers to families of inner-city children with asthma, during an acute hospital admission, was associated with improved asthma management behaviours [24].

Our qualitative sub-study sampled the experiences of a range of participants involved in the asthma education sessions, to facilitate triangulation of our findings. One limitation of our study could be that those agreeing to participate may have had a more positive view of the intervention; however, participants were purposively sampled with only two potential participants declining to take part, with the main reason given being time constraints. To ensure that participants felt comfortable to freely express their opinions, FGDs and interviews were facilitated by an independent researcher, with no previous connection to the RCT participants.

## Conclusion

In conclusion, asthma education delivered by lay educators was well received by children and their families, with reported positive benefits on asthma knowledge levels, symptoms, and daily life, and increased confidence relating to asthma self-management. Training, support and motivation are essential facilitators that must be present to successfully implement such an intervention. There is a need to evaluate the wider implementation of this approach, beyond a tertiary hospital setting, and to explore optimal ways to train, support and motivate lay educators in LMIC settings. We predict the impact of such an intervention may be greater in LMICs than in HICs due to shortages of healthcare staff and lower health literacy levels. If Universal Health Coverage is to be achieved, low-income settings must find more affordable and efficient ways to deliver the increasing burden of care relating to NCDs—carefully implemented reallocation of tasks to non-clinical staff is a potential way to realise this goal [25].

## Supplementary Information

**Additional file 1.** A description of the Lay educators and the asthma education intervention.

## Data Availability

Data and materials are available from the corresponding author (LN: ORCID ID: 0000-0002-1457-6022) upon reasonable request.

## Abbreviations

LMICs: Low-middle-income countries
QECH: Queen Elizabeth Central Hospital
GINA: Global initiative for asthma
HICs: High income countries
RCT: Randomised control trial
FGDs: Focus group discussions
SSA: Sub-Saharan Africa
