# Supplementary material for: Task-shifting to improve asthma education for Malawian children: a qualitative analysis
Source: Hum Resour Health. 2021 Mar 2;19:28. doi: 10.1186/s12960-021-00576-1 (PMC7927223; doi:10.1186/s12960-021-00576-1)
Supplement: Supplementary file 1 — Additional file 1. A description of the Lay educators and the asthma education intervention. [file 12960_2021_576_MOESM1_ESM.docx]

**ADDITIONAL FILE 1**

**Asthma education intervention**

***Pre-study training***

Before the study started, four “lay educators” received training by the study doctor. These staff had completed secondary school, with a Malawi School Certificate of Education, but had no previous medical education experience or training. All were employed as “fieldworkers” with the Malawi-Liverpool-Wellcome Trust Clinical Research Programme and had received spirometry training (Certificate of Competence in Foundational Spirometry) on their previous projects. The lay educators spent time with the study doctor in the paediatric clinic to gain experience with asthma patients and their families. Formal training comprised four hour-long tutorials (Table S1), with an accompanying training manual for self-study, after which the educators were required to gain at least 80% in a knowledge test (comprising 20 true/false questions) and demonstrate competence at delivering the asthma education session in a role-play session. The educators were supported by the study medical

staff and encouraged seek advice throughout the study period.

Table S1. Pre-study asthma training for lay educators

| Session | Topic | Key content |
| --- | --- | --- |
| 1 | What is asthma | Chronic inflammation of airways and airway narrowing Recurrent symptoms; cough, wheeze, difficulty breathing Impact of poor asthma control, including death with severe attack  Symptoms can be well controlled with inhaled treatment  Common triggers for asthma symptoms |
| 2 | Asthma treatment | Types of inhaler: relievers (β2 agonist) and preventers (steroid)  Use of a spacer to improve drug delivery  Importance of long-term adherence |
| 3 | Self-management | Monitoring of symptoms  What to do in an asthma attack  Asthma Action Plans |
| 4 | Practical session | Inhaler technique – how to demonstrate  How to deliver an asthma education session |

**Education session content**

Study participants received a 1-hour individualised asthma education session, delivered to the child and their carer by lay educators, with oversight by the study doctor or nurse. A structured approach, with checklist (Table S2), was followed to ensure intra- and inter-educator consistency. Education sessions were conducted in Chichewa, and patients received a written asthma action plan, also in Chichewa.
